# Supplementary material for: Patterns of Intron Gain and Loss in Fungi
Source: PLoS Biol. 2004 Nov 30;2(12):e422. doi: 10.1371/journal.pbio.0020422 (PMC532390; doi:10.1371/journal.pbio.0020422)
Supplement: Table S1 — Also available at http://genes.mit.edu/NielsenEtAl/. (4.3 MB ZIP). [file pbio.0020422.st001.zip › NielsenEtAl/html/657.html]

AN9526.1.NCU01907.1.MG02920.1.FG08940.1


```
 CLUSTAL W (1.82) Multiple Sequence Alignments - Introns Inserted


Sequence 1: MG02920.1	329 aa
Sequence 2: FG08940.1	321 aa
Sequence 3: NCU01907.1	317 aa
Sequence 4: AN9526.1	344 aa
Alignment Length: 345 aa
Number Identitical Residues: 192 aa
Alignment Score (without introns) 8317


MG02920.1 	MAVASVQDRTAEFKSVLAQAQKRQANAKASAQRRSLLTDAQKADANG-------APPRRS
NCU01907.1	MAVFAINDRTEEFRQIVAAAQRRQA-TKPGKQR--LLDTAQQHAASS------DAQPRRS
FG08940.1 	MAVASIQDRTSEFKSVLAQAQRKQTSSKVSSQRRSLLTDAQKDAANGHP----TGPPRRS
AN9526.1  	MTGPTIQDRTSEFQAILGQAQKRAASSKVGSQRQALLTDSQRQQANGSPNGTAAGGKRRS
          	*:  :::*** **: ::. **:: :.:* . ** :**  :*:  *.. ...:: .  ***

MG02920.1 	EFARRAAEIGRGIAATMGKLEKLAQL~AKRKTLFDDRPVEINE~LTFIIKQDLSSLNQQI
NCU01907.1	EFARGAAEIGRGISATMAKLEKLAQL~AKKKTLFDDRPVEINE0LTFVIKQDLSSLNEKI
FG08940.1 	DFARKAAEIGRGISATMGKLEKLAQL1AKRRTLFDDRPVEINE~LTFVIKQDLSSLNQQI
AN9526.1  	EFARRAAEIGRGITATTAKLRRLAEL1AKRKTLFDDRPVEISE~LTYVIKQDLASLNQQI
          	:*** ********:** .**.:**:* **::**********.* **::*****:***::*

MG02920.1 	SGLQQLSRQQHPKA-----DQEGEHNKN~VVFLLQGKLTDVSANFKDVLEVRTKNIQASR
NCU01907.1	RNLQDLSRRLHPKP-----DQEGENNKN~ILLLLQGKLGDVGANFKDVLEIRTKNIQASR
FG08940.1 	GALQTITKQQHPKA-----DQEGEHNKN~VVYLLQGKLTDVSVNFKDVLEARTKNIQASR
AN9526.1  	ASLQALTLSQHPKSNRSKTDQEGEHNDN0VVVMLQGKLADVGANFKDVLEVRTKNIQASR
          	  ** ::   ***.. :.:*****:*.* :: :***** **..******* *********

MG02920.1 	SRTENFISSVSQHAQQ-PVLQSSASPLYGTPNRGTPSPGV--DLLTLNPPGGKGMGG-GP
NCU01907.1	SRTENFVSSVGQHAH--ASLQQSASPLYGTPSRGTPAPGQQ-DLISLN-----------P
FG08940.1 	SRTENFISSVSQHAQ--PSIQQSASPLYGTPARNSPAPGQ--DTLSLN-----------P
AN9526.1  	SRTENFVSSVSSKSQAALDPQRSDSPLYPS-GRRTPQPGGSSDLLTLEPSNPSPLGRPSM
          	******:***..::: :   * * **** :  * :* ** .:* ::*:... .  . .. 

MG02920.1 	VGDEQLMLMEEAQPQNAYIQQRGEAIEAIEKTIAELGGIFGQLAGMVSEQSEMIQRIDAN
NCU01907.1	MGDQQMQLQMLEEGQNTYVQQRGQAIEAIESTINELGSIFGQLAAMVSEQSEMIQRIDAN
FG08940.1 	VGDQQLLMMEEAQPSNTYIQQRGEAIEAIEKTIGELGSIFGQLATMVSEQSEMIQRIDAN
AN9526.1  	QSDQQLLMMEEAESSNSYIQSRGEAIDAIERTINELGGIFGQLAQMVSEQSEMIQRIDAN
          	 .*:*: :    : .*:*:*.**:**:*** ** ***.****** ***************

MG02920.1 	T~EDVVDNVEGAQRELLKYWSRVSGNRMLIAKMFGVL~M~IFFL2LWVLIAG
NCU01907.1	T2EDVVENVEGAQKELLKYWSRVSSNRWLLAKMFGVL0M~IFFL~LWVLIAG
FG08940.1 	T~EDVVDNVEGAQRELLKYWNRVSSNRWLIAKMFGVL~M~IFFL2LWVLVSG
AN9526.1  	T~EDVVDNVQGAQRELMKYWTRVSGNRWLIAKMFGVL~M0IFFL~LWVLISG
          	* ****:**:***:**:***.***.** *:******* * **** ****::*
```
